# ASSESSING READINESS OF BLACK OLDER ADULTS TO DISCUSS ADVANCE CARE PLANNING WITH THEIR SURROGATES

**DOI:** 10.1093/geroni/igae098.3794

**Published:** 2024-12-31

**Authors:** Ekenechukwu Akabike, Rebecca Howe, Alicia Agnoli, Julie Bidwell, Grace Amadi

**Affiliations:** University of California, Davis, Sacramento, California, United States; VA Providence Healthcare System, Providence, Rhode Island, United States; University of California, Davis, Sacramento, California, United States; University of California, Davis, Sacramento, California, United States; University of California, Davis, Sacramento, California, United States

## Abstract

Advance care planning (ACP) is a tool to identify health care goals in serious situations or if anticipating end-of-life circumstances. ACP is not always utilized by those who might most benefit from it and appears underutilized by black older adults for unclear reasons. This study explores 3 aims to better understand the readiness of black older adults to complete ACP including examining patient readiness to participate in ACP using the stages of change model, types of barriers and promoting factors to patient participation in ACP, and patients and surrogates’ concordance on ACP readiness. A sub-analysis explores how readiness for ACP participation varies based on the patient-surrogate relationship. A mixed methods design used surveys and qualitative interviews to assess black older adults aged 60 – 91 and their associated surrogate selected from outpatient clinics within an academic hospital and a local community church in Sacramento, CA. Participants included 30 older adult respondents with 12 surrogates consisting of 7 adult children and 5 spouses/partners. Results suggest variation of end-of-life care discussion readiness where 63% of surrogates report already completing this discussion but only 45% of patients with surrogates report similar findings. Variation also occurs with patient readiness to discuss end-of-life care with adult children (17% of patients with children surrogates report not thinking about having these discussions yet) compared to those with a spouse/partner surrogate (100% of patients with partners/spouses have already had discussions). Additional research may help increase understanding of themes of patient and surrogate readiness and engagement in ACP.

